# Enhancing segmentation accuracy of the common iliac vein in OLIF51 surgery in intraoperative endoscopic video through gamma correction: a deep learning approach

**DOI:** 10.1007/s11548-025-03388-z

**Published:** 2025-05-11

**Authors:** Kaori Yamamoto, Reoto Ueda, Kazuhide Inage, Yawara Eguchi, Miyako Narita, Yasuhiro Shiga, Masahiro Inoue, Noriyasu Toshi, Soichiro Tokeshi, Kohei Okuyama, Shuhei Ohyama, Satoshi Maki, Takeo Furuya, Seiji Ohtori, Sumihisa Orita

**Affiliations:** 1 Department of Medical Engineering, Faculty of Engineering, Chiba University, 1-33 Yayoi-cho, Inage-ku, Chiba, 263-8522 Japan; 2 Faculty of Medicine, Nara Medical University, 840 Shijo-Cho, Kashihara, Nara, 634-8521 Japan; 3 Department of Orthopaedic Surgery, Graduate School of Medicine, Chiba University, 1-8-1 Inohana, Chuo-ku, Chiba, 260-8670 Japan; 4 Department of Orthopaedic Surgery, Shimoshizu National Hospital, 934-5, Shikawatashi, Yotsukaido, Chiba, 284-0003 Japan; 5 Center for Frontier Medical Engineering, Chiba University, 1-33 Yayoi-cho, Inage-ku, Chiba, 263-8522 Japan

**Keywords:** Oblique lateral lumbar interbody fusion (OLIF) 51, Segmentation, Deep learning, Vein segmentation, Preprocessing, Gamma correction

## Abstract

**Purpose:**

The principal objective of this study was to develop and evaluate a deep learning model for segmenting the common iliac vein (CIV) from intraoperative endoscopic videos during oblique lateral interbody fusion for L5/S1 (OLIF51), a minimally invasive surgical procedure for degenerative lumbosacral spine diseases. The study aimed to address the challenge of intraoperative differentiation of the CIV from surrounding tissues to minimize the risk of vascular damage during the surgery.

**Methods:**

We employed two convolutional neural network (CNN) architectures: U-Net and U-Net++ with a ResNet18 backbone, for semantic segmentation. Gamma correction was applied during image preprocessing to improve luminance contrast between the CIV and adjacent tissues. We used a dataset of 614 endoscopic images from OLIF51 surgeries for model training, validation, and testing.

**Results:**

The U-Net++/ResNet18 model outperformed, achieving a Dice score of 0.70, indicating superior ability in delineating the position and shape of the CIV compared to the U-Net/ResNet18 model, which achieved a Dice score of 0.59. Gamma correction increased the differentiation between the CIV and the artery, improving the Dice score from 0.44 to 0.70.

**Conclusion:**

The findings demonstrate that deep learning models, especially the U-Net++ with ResNet18 enhanced by gamma correction preprocessing, can effectively segment the CIV in intraoperative videos. This approach has the potential to significantly improve intraoperative assistance and reduce the risk of vascular injury during OLIF51 procedures, despite the need for further research and refinement of the model for clinical application.

## Introduction

**Fig. 1:**
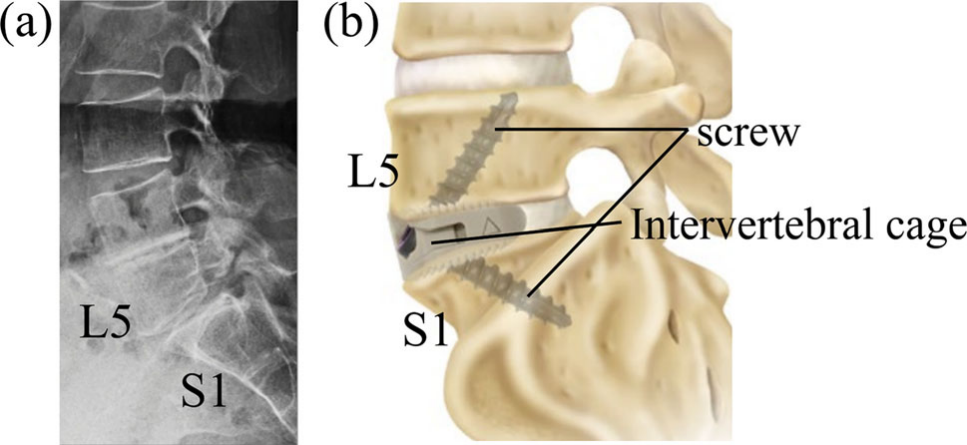
**a** Representative X-ray image of spondylolisthesis: L: lumbar, S: sacrum. **b** Post-operative image of OLIF51 using intervertebral Cage fixed with vertebral fixation screws

Oblique lateral interbody fusion for L5/S1 (OLIF51) is a minimally invasive anterior spinal fusion technique performed for degenerative lumbosacral spine diseases such as lumbar spondylolysis and lumbar spondylolisthesis (Fig. 1a). The procedure exposes the anterior portion of the L5/S1 intervertebral disk (IVD) in a small field of view and inserts an intervertebral cage and screws for fixation to achieve anterior fixation (Fig. 1b) [1, 2].

**Fig. 2:**
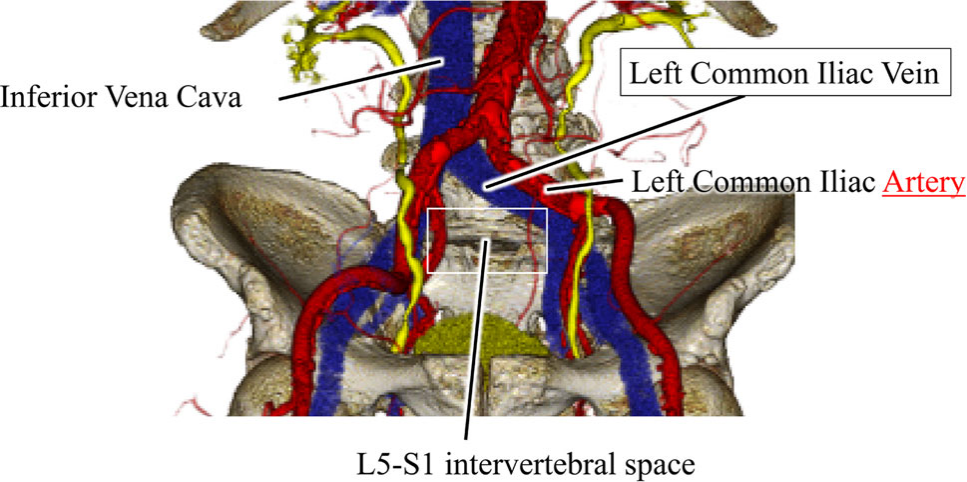
Vascular anatomy around the L5/S intervertebral area. The area enclosed by a square indicates the site approached during OLIF51 surgery. Among the left and right common iliac veins (CIV) branching from the inferior vena cava, particularly the left CIV runs near the surgical field. Intraoperative injury to the left CIV can lead to fatal bleeding

The procedure approaches the L5/S1 intervertebral disk from the anterior side of the psoas major in the right lateral decubitus position (Fig. 2). When advancing the surgical field to the L5/S1 IVD from the major vascular bifurcation, the common iliac vein (CIV) anatomically runs close to the surgical site. Damage to CIV can result in fatal massive hemorrhage, with the rate of vascular damage caused by retractors and surgical instruments reported to be between 4.1 and 4.3% [1, 3]. To deal with the complications, intraoperative endoscopy is helpful to ensure the accurate positioning of the surgical field and surrounding tissues, including the veins, and to share with external parties. On the other hand, CIV often runs behind the peritoneum and arteries, and the color tone of the surrounding tissues is sometimes difficult to identify, making differentiation difficult. Furthermore, the position of the vein is compared with the actual surgical field based on preoperative MRI (Magnetic resonance image) or CT (Computed tomography) 3D reconstruction images. The recognition of the vein position can rely heavily on the surgeon’s experience, meaning that the use of an assistive endoscope does not necessarily translate directly to a reduced risk of damage. Therefore, appropriate intraoperative support for this procedure is required, but no such support system is currently established.

To solve this problem, AI (Artificial Intelligence) can be helpful, and that has been utilized in various fields, especially in the medical field, where the introduction of AI is expected for medical image diagnosis, prognosis prediction, and robot control [4]. Deep learning is a machine learning method that uses neural networks modeled after the human brain’s structure. Deep learning can flexibly extract features associated with complex data, enabling image and voice recognition, natural language processing, and more. Image recognition has been used for medical image analysis, and a wide range of research is being conducted on segmentation tasks and object detection [5]. Segmentation tasks label each pixel in the image and classify semantic regions. Related research has recorded high accuracy in segmenting blood vessels and polyps [6, 7]. The introduction of AI in the medical field is expected to greatly assist physicians by significantly reducing examination time, reducing the burden on physicians, reducing the risk of oversight, and compensating for differences in the experience of physicians, who are primarily responsible for making decisions [8]. Therefore, the presentation of the total iliac vein segmentation in OLIF51 can ensure the safety of the surgical field.

The current study aimed to develop and evaluate a model that uses deep learning to segment the common iliac vein from intraoperative endoscopic videos of OLIF51.

## Materials and methods

### Semantic segmentation methods

Semantic segmentation is different from object detection, which obtains positional information at the rectangular unit level; it can distinctly detect objects of any shape. This is highly practical for medical image processing applications that require detailed image mapping. In this paper, we utilize the architectures of U-Net and U-Net++ and employ ResNet18 as the backbone feature extractor in the convolutional neural network, evaluating the accuracy with these two models [9–11]. U-Net is a convolutional neural network (CNN) introduced in 2015 by Olaf Ronneberger et al., intended for biomedical image segmentation [9]. It features a skip-connection technique that connects the feature maps of the encoder to those of the decoder, making it easier to capture the positional information of objects. U-Net++ was developed by Zongwei Zhou et al. as a more robust architecture for biomedical segmentation [10]. Compared to U-Net, U-Net++ has denser connections between the encoder and decoder, aimed at reducing the gap between the feature maps of the subnetworks. ResNet is a convolutional neural network proposed in 2015 by a team from Microsoft Corporation [11]. Its characteristic is the ability to avoid the gradient vanishing problem, where gradients diminish and disappear as they backpropagate through the layers, enabling the learning of deeper networks.

The development method for the total iliac vein segmentation model is explained. Endoscopic video frames are inputted into the network, and the vein segmentation inference mask for the frames is overlaid onto the input images as the output. The parameters and loss functions used for training are listed in Table 1.

**Table 1:** Training detail

epoch	50
batch_size	32
learning_rate	0.0001
optimizer	Adam
Loss function	DiceLoss

### Dataset

**Fig. 3:**
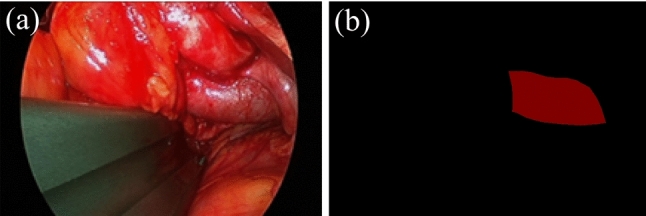
Pairs of endoscopic images (unprocessed) and correct masks; **a** endoscopic image showing common iliac vein (CIV) and **b** Correct masks

The dataset was created using intraoperative endoscopic videos from four cases of OLIF51 provided by the Department of Orthopedics at our facility. The videos were split every 15 frames, and only images showing the total iliac vein were used to create the dataset. A total of 614 images were used: 401 for training (2 cases), 168 for validation (1 case), and 45 for testing (1 case). The open annotation tool LabelMe was used to create the correct masks. Segmentation was performed by placing polygons around the object, and the boundaries of the object were precisely specified manually in each frame. This was done because we determined that manual polygon placement was the best way to specify the exact anatomical boundaries of the object site. In addition, by doing it completely manually, we were able to accurately reflect microscopic structures and complex shapes without overlooking them. Although one evaluator was responsible for label creation, the work was performed under the guidance of a medical specialist, and the mask images were verified by a clinician. Figure 3 shows pairs of endoscopic images (unprocessed) and correct masks actually used for training.

### Preprocessing consideration

CIV and the surrounding tissue belong to the red color system in the hue spectrum. They are difficult to distinguish at the pixel level due to similar saturation lightness and low contrast.

In this study, to emphasize the luminance difference between veins and surrounding tissues due to their anatomical and visual characteristics, gamma correction was applied to the R (Red) channel in image preprocessing. Gamma correction is a process of arbitrarily converting the image’s luminance. The goal is to further emphasize the difference in color between surrounding tissues and arteries, making the features more prominent. The formula for gamma correction is shown in Eq. 1.

2 $$f\left(x\right)={\left(\frac{x}{255}\right)}^{ \frac{1}{\gamma }}\times 255 $$

**Fig. 4:**
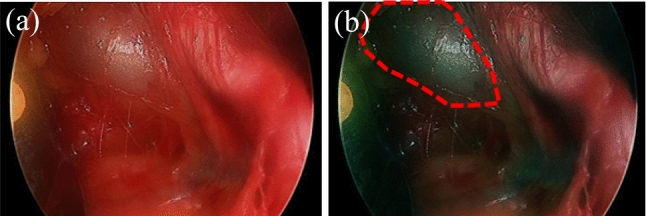
The changes in the images due to gamma correction: **a** endoscopic image before correction and **b** endoscopic image after correction

This γ value was set to 0.35 to increase the luminance difference. The reason for fixing the gamma value at 0.35 is that the gamma coefficient was changed in advance from 0.1 to 0.9 in 0.05 steps, and sthe training results showed the highest accuracy at 0.35. Figure 4 illustrates the changes in the images due to gamma correction. The red-dotted line in Fig. 4b surrounds the vein, and it is visually confirmed that the color difference of the vein is emphasized compared to the artery on the right and the pre-correction image in Fig. 4a. It is hypothesized that this difference is also effective in learning, and a comparison of accuracy with and without correction is performed. As a method of validating the effectiveness of the gamma correction, the Wilcoxon test for the test data was used to evaluate it. The Wilcoxon test is a nonparametric test to determine if the difference between their medians in two related datasets is statistically significant. This test does not assume that the data follow a normal distribution and are therefore less sensitive to the shape of the distribution.

The purpose of the Wilcoxon test in this study is to confirm that the difference in model performance (e.g., recall and precision) with and without gamma correction is significant and not due to chance.

### Evaluation metrics

Evaluation metrics used in the semantic segmentation task, such as dice, recall, precision, and specificity, were used. SSIM (Structural similarity index measure) and PSNR (Peak signal-to-noise ratio) were used to evaluate the structural similarity of the images.

## Results

### Segmentation methods

**Fig. 5:**
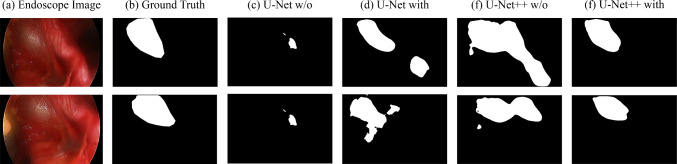
Segmentation comparison by model: **a** endoscope image, **b** ground truth, **c** U-Net w/o, **d** U-Net with, **e** U-Net++ w/o, and **f** U-Net++ with gamma correction

A total of four models were created for the U-Net and U-Net++ models, with and without gamma correction. The dice of each model with the test data is shown in Table 2. The comparison of segmentation inference masks by each model is shown in Fig. 5. In this study, the gamma-corrected U-Net++ model showed the best results in several evaluation metrics, including recall, precision, specificity, SSIM, and PSNR. In particular, the best model showed higher values for both recall and precision, indicating fewer false positives (overdetections) and fewer false negatives (underdetections). On the other hand, there were no significant differences among all models with respect to PSNR. This is due to the fact that PSNR is an index that calculates accuracy based on pixel values, and since the images used in this study were binary black-and-white images, it is difficult to reflect minute differences. As a result, the PSNR values were nearly equal among the models.

**Table 2:** The dice of each model with the test data

Model	Gamma correction	Dice	Recall	Precision	Specificity	SSIM	PSNR
U-Net[9]	w/o	0.39	0.65	0.32	0.93	0.90	12.47
	with	0.59	0.66	0.65	0.99	0.96	16.86
U-Net++[10]	w/o	0.44	0.45	0.62	0.99	0.96	16.16
	with	0.70	0.74	0.77	0.99	0.97	18.99

Regarding segmentation accuracy, with gamma correction, the model with U-Net++ architecture and ResNet18 backbone yielded the best results. U-Net++ was able to roughly capture the position and shape of the veins, recording a dice score of 0.70, while U-Net was inferior in capturing shape compared to U-Net++, resulting in a dice score of 0.59. In addition, the accuracy of each model was inferior without gamma correction.

### Effectiveness of gamma correction

**Fig. 6:**
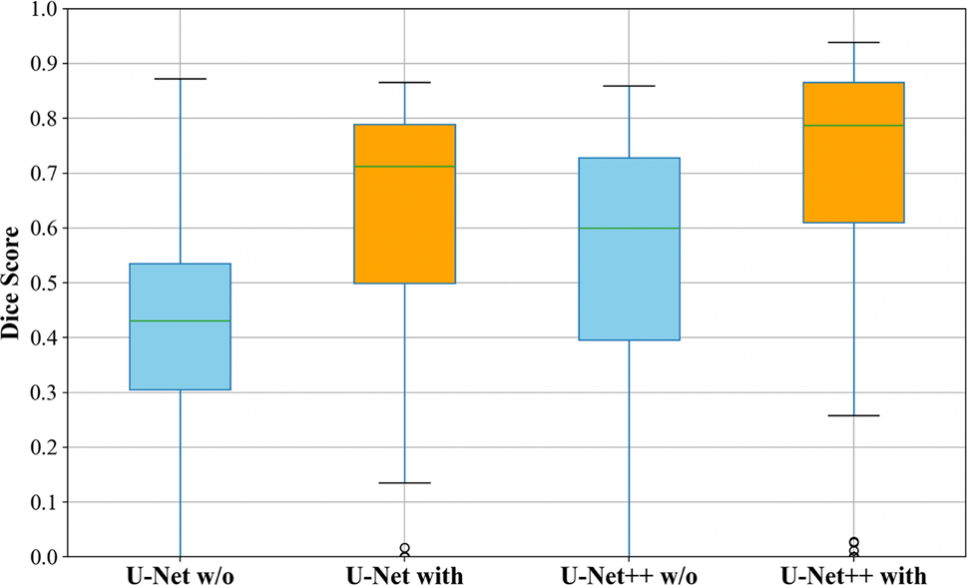
Comparison of dice scores for different models on test data. Box-and-whisker diagram showing the distribution of dice scores for each model evaluated in the test data set. The horizontal line represents the mean and the boxes represent the interquartile range (IQR). As a result, the U-Net++ model with correction achieved the highest median dice score. In addition, the models with each model correction tended to have lower variance than the models without correction

Figure 5c and e shows the segmentation results without gamma correction, while (d) and (f) shows the segmentation results with gamma correction. In images without gamma correction, the artery on the right is included in the segmentation, whereas in images with gamma correction, only the vein is segmented. The segmentation accuracy without correction had an average dice score of 0.44 for the U-Net++ model, and more than half of the test data included arteries in the segmentation. However, as shown in Fig. 5, the model with correction did not detect arteries and further demonstrated higher accuracy. The distribution of dice scores for each model is shown in Fig. 6. Models without gamma correction are shown in light blue, and models with gamma correction are shown in orange. Similar to the results in Table 2, Fig. 6 shows that gamma correction increases the overall distribution of dice scores. It is also confirmed that the gamma correction suppresses the variability of the dice score. A paired-sample Wilcoxon signed-rank sum test was conducted on the dice scores of the U-Net++/ResNet18 model to see if there was a significant difference between the two models with and without gamma correction. The results were Z = −10.83 and *p* = 2.56e^−27^, indicating a significant difference due to the gamma correction process.

**Fig. 7:**
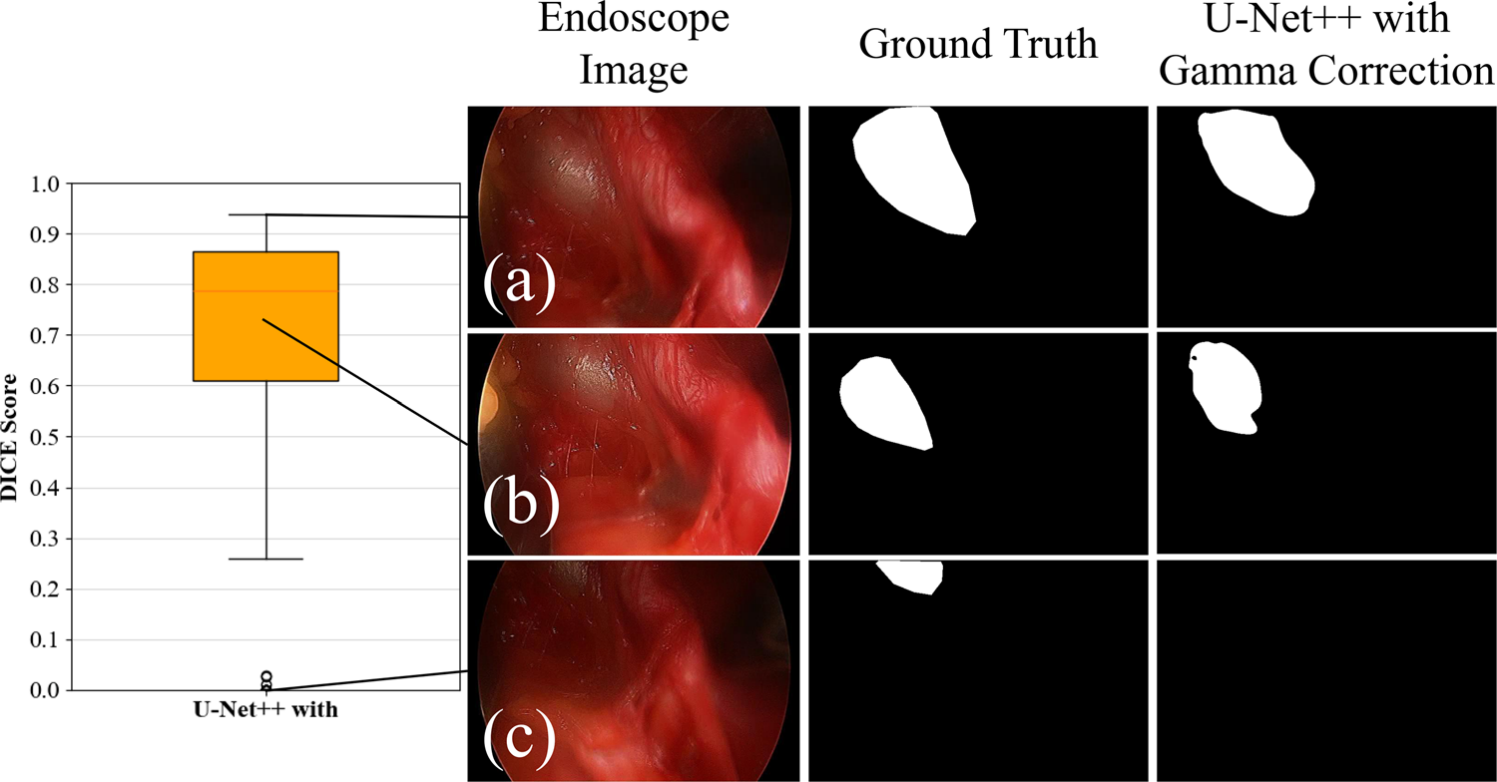
Dice distribution and segmentation results for the best model (U-Net++ with Gamma correction). Dice coefficients are **a** 0.94, **b** 0.73, and **c** 0, respectively

Regarding the best model, it could approximately capture the position and shape of the vein better than other models. Figure 7a shows the distribution of test data evaluations for the best model. Although several outliers have results below 0.30, most recorded accuracies fall between 0.7 and 0.8. The highest accuracy recorded was 0.94. Figure 7b shows the segmentation results of the best model. When the entire total iliac vein fits within the frame, it generally captures the shape of the vein. However, accuracy greatly decreases when the vein is cutoff from the frame or when the full view is unclear because the light source does not reach the deep parts.

## Discussion

The OLIF51 procedure, a minimally invasive anterior spinal fixation technique, often presents a challenge in distinguishing the CIV due to its color similarity with surrounding tissues. Currently, there is no established intraoperative support system for this specific procedure.

In the current study, we have created and evaluated a deep learning model for segmenting the CIV, recording a Dice coefficient of 0.59 with the U-Net/ResNet18 model and 0.70 with the U-Net++/ResNet18 model.

Comparisons with similar studies are as follows: A vascular segmentation study using the architecture NephCNN recorded a dice coefficient of 0.72 [6]. Another study conducted semantic segmentation on gastric polyps in gastroenterology using the architecture U-Net++ with ResNet50 as the backbone, which recorded a dice coefficient of 0.98 [7]. Compared to the former study of vessel segmentation, the accuracy of dice in this study is comparable to that of the former study. This suggests that this method is at least effective in improving intraoperative navigation accuracy. The latter study on polyp segmentation has achieved an average evaluation close to 100% with a dice coefficient of 0.98. In comparison, the segmentation accuracy of our study is considerably lower. Although our study can be considered a novel solution for assistance during the OLIF51 procedure, further improvements in the model’s reliability are necessary for enhanced accuracy.

We are developing more improvements to the model to enhance accuracy. In our study, we input one frame at a time to the model, which loses the advantage of continuous video frames. LSTM (Long short term memory) and GRU (Gated recurrent unit) are networks suitable for handling time-series data and can incorporate past information into the current state [12, 13]. Research has shown that accuracy improves when using these networks or combining them with CNNs compared to using simple CNNs alone [14, 15]. By combining these networks, tracking the position and shape of the vein through frames can further enhance accuracy.

Regarding the effectiveness of gamma correction, the segmentation accuracies recorded were a dice coefficient of 0.44 without correction and 0.70 with correction. In previous studies, the contrast between the vein and surrounding tissues was low, and variations in color tone and course among individuals were challenges for segmenting internal tissues like the total iliac vein. Under the hypothesis that gamma correction makes the veins visually distinct from surrounding tissues, our method proceeded with experiments and confirmed through accuracy evaluation that the corrected cases achieved higher accuracy than the uncorrected ones. In particular, Fig. 5 shows that only veins are specifically detected. The distribution of dice scores in Fig. 6 shows that the correction not only improved segmentation accuracy but also suppressed numerical variability. This suggests that feature extraction through gamma correction is effective for vein segmentation. Similar to our method, reports of distinguishing veins from surrounding tissues through gamma correction have not been conducted within the scope of our research, and it can be considered an advantage of our study. Increasing the number of cases and conducting further tests will be effective going forward.

This study has several limitations. Firstly, the small dataset size impacts the robustness of our model, especially given that deep learning models benefit from larger training datasets. Secondly, individual variations in the color and position of the iliac vein require a more diverse dataset to overcome contrast issues. Also, one evaluator was in charge of label creation, but there are certain limits to the consistency among the evaluators. In the future, we plan to increase the number of evaluators to further improve the reliability of the labels and to incorporate periodic reviews by physicians to improve accuracy. Lastly, the current standards for facilities and surgeons performing OLIF51 surgeries may limit the model’s clinical applicability and safety assurance.

## Conclusion

We developed and evaluated a model for segmenting the total iliac vein using deep learning from intraoperative endoscopic videos of OLIF51 procedures. Regarding segmentation methods, the architecture U-Net++ combined with the backbone ResNet18 model recorded the best average Dice score of 0.70. The U-Net++ architecture demonstrated higher accuracy than U-Net. Furthermore, feature extraction through gamma correction was suggested to be effective for segmenting veins. This technology could significantly contribute to intraoperative assistance during OLIF51 procedures.

## References

[1] Kamal W, James B, Richard H (2017) Technical description of oblique lateral interbody fusion at L1–L5 (OLIF25) and at L5–S1 (OLIF51) and evaluation of complication and fusion rates. Spine J 17:545–553. 10.1016/j.spinee.2016.10.026 27884744 10.1016/j.spinee.2016.10.026

[2] Orita S, Kazuhide I, Takeo F, Masao K, Yasuchika A, Go K, Junichi N et al (2017) Oblique lateral interbody fusion (OLIF): indications and techniques. Oper Tech Orthop 27:223–230. 10.1053/j.oto.2017.09.004

[3] Hah M, Myeong K, Young K, Seung P (2020) Usefulness of oblique lateral interbody fusion at L5–S1 level compared to transforaminal lumbar interbody fusion. J Korean Neurosurg Soc 63:723–729. 10.3340/jkns.2018.0215 31295977 10.3340/jkns.2018.0215 PMC7671784

[4] Andre E, Alexandre R, Bharath R, Volodymyr K, Mark D, Katherine C, Claire C, Greg C, Sebastian T, Jeff D (2019) A guide to deep learning in healthcare. Nat Med 25:24–29. 10.1038/s41591-018-0316-z 30617335 10.1038/s41591-018-0316-z

[5] Xuxin C, Ximin W, Ke Z, Kar-Ming F, Theresa C, Kathleen M, Robert S, Hong L, Bin Z, Yuchen Q (2020) Recent advances and clinical applications of deep learning in medical image analysis. Med Image Anal 79:102444. 10.1016/j.media.2022.102444 10.1016/j.media.2022.102444 PMC9156578 35472844

[6] Alessandro C, Sara M, Chiara C, Emanuele F, Elena M, Leonardo S (2021) NephCNN: A Deep-Learning Framework for Vessel Segmentation in Nephrectomy Laparoscopic Videos. In: 2020 25th International Conference on Pattern Recognition, 6144–49. 10.1109/ICPR48806.2021.9412810

[7] Roi N, Issa N, Dror R, Mustafa Y, David A (2023) Segmentation of polyps based on pyramid vision transformers and residual block for real-time endoscopy imaging. J Pathol Inform 14:100197. 10.1016/j.jpi.2023.100197 36844703 10.1016/j.jpi.2023.100197 PMC9945716

[8] Tejal K, David C, Christine A, David C, Nikki G, Andrew R, Frank F (2023) How can artificial intelligence decrease cognitive and work burden for front line practitioners? JAMIA Open 6:ooad079. 10.1093/jamiaopen/ooad079 37655124 10.1093/jamiaopen/ooad079 PMC10466077

[9] Olaf R, Philipp F, Thomas B (2015) U-Net: Convolutional Networks for Biomedical Image Segmentation. Medical Image Computing and Computer-Assisted Intervention—MICCAI 2015. Lecture Notes in Computer Science, vol 9351. Springer, Cham, pp 234–241. 10.1007/978-3-319-24574-4_28

[10] Zongwei Z, Mahfuzur S, Nima T, Jianming L (2018) UNet++: A Nested U-Net Architecture for Medical Image Segmentation. Deep Learn Med Image Anal Multimodal Learn Clin Decis Support, pp 3–11. 10.1007/978-3-030-00889-5_1 10.1007/978-3-030-00889-5_1 PMC7329239 32613207

[11] Kaiming H, Xiangyu Z, Shaoqing R, Jian S (2016) Deep Residual Learning for Image Recognition. 2016 IEEE Conference on Computer Vision and Pattern Recognition (CVPR), pp 770–78. 10.1109/CVPR.2016.90

[12] Sepp H, Jürgen S (1997) Long short-term memory. Neural Comput 9:1735–80. 10.1162/neco.1997.9.8.1735 9377276 10.1162/neco.1997.9.8.1735

[13] Junyoung C, Caglar G, KyungHyun C, Yoshua B (2014) Empirical Evaluation of Gated Recurrent Neural Networks on Sequence Modeling. In: Proceedings of the 27th International Conference on Neural Information Processing Systems (NIPS 2014), vol 2, pp 1786–1794. https://nyuscholars.nyu.edu/en/publications/empirical-evaluation-of-gated-recurrent-neural-networks-on-sequen

[14] Lirong Y, Lei W, Tingqiao L, Siyu L, Jiawei T, Zhengtong Y, Xiaolu L, Wenfeng Z (2023) U-Net-LSTM: time series-enhanced lake boundary prediction model. Land 12:1859. 10.3390/land12101859

[15] Eisuke S, Kazuhiro H (2022) Cell image segmentation by using feedback and convolutional LSTM. Vis Comput 38:3791–3801. 10.1007/s00371-021-02221-3

